# Modified Surgical Stent in Management of Odontogenic Cyst in Mixed Dentition: A Report of Two Cases

**DOI:** 10.30476/DENTJODS.2021.88630.1353

**Published:** 2022-06

**Authors:** Shrirang Anand Sevekar, Sunil Omprakash Sidana, Subhadra Halemane Nagraj

**Affiliations:** 1 Dept. of Pediatric & Preventive Dentistry, MGM Dental College & Hospital, Navi Mumbai, Maharashtra, India; 2 Dept. of Oral & Maxillofacial Surgery, MGM Dental College & Hospital, Navi Mumbai, Maharashtra, India; 3 Dept. of Pediatric & Preventive Dentistry, YMT Dental College & Hospital, Navi Mumbai, Maharashtra, India

**Keywords:** Odontogenic cysts, Decompression, Surgical, Dentition, Mixed, Splints, Space maintenance, Orthodontics

## Abstract

The treatment choice in the management of odontogenic cysts in the mixed dentition period depends upon the size, location of the cyst, the bone integrity of the cystic wall, and its proximity to vital structures. Enucleation is indicated with smaller cysts, achieved by careful removal of a complete cyst without rupturing the cystic lining. Marsupializationand decompression are the treatments of choice for larger cysts as it can help to preserve the tooth bud of the successor tooth and reduce morbidity. Marsupialization is achieved by opening and deroofing the cyst, and making the cystic lining continuous with the oral cavity or surrounding structures by suturing the edges of the incised mucosa to the cystic wall. This helps in maintaining the patency of the cystic lesion. In the decompression, a cylindrical device (drain) is placed in the lesion, which maintains communication between oral cavity to cystic lesion. This decreases the intracystic pressure and results in bone formation. We present two cases of odontogenic cyst in children, where we used a modified decompression technique. We developed a modified surgical stent with the use of a Hawley’s appliance, which led to cystic decompression, and eventual eruption of the successor tooth. Notably, this modified technique also reduced both patient discomfort and the number of clinical visits, making it an effective treatment option. The unique design of the appliance also acted as a space maintainer for the eruption of successor tooth, which is very critical in mixed dentition for future prevention of space loss and eventual malocclusion. The advantage of our design was its easy removal and insertion with minimal discomfort.

## Introduction

Enucleation, marsupialization, and decompression are the treatments of choice for the management of odontogenic cysts. Enucleation consists of the careful removal of a complete cyst without rupturing the cystic lining, particularly indicated with smaller cysts. With larger cysts, however, enucleation can lead to mandible fracture, devitalisation of the adjacent teeth, and amputation of the successor tooth buds suggesting marsupialization and decompression are the more viable options [ 1 ]. Marsupialization is achieved by opening and deroofing the cyst, and making the cystic lining continuous with the oral cavity or surrounding structures by suturing the edges of the incised mucosa to the cystic wall. This helps in maintaining the patency of the cystic lesion. Sometimes, medicated gauze is inserted into the cystic lesion to prevent its spontaneous closure [ 2 - 3 ]. In the decompression technique, a drain is placed in the lesion, which maintains communication between oral cavity to cystic lesion. This decreases the intracystic pressure and results in bone formation [ 4 ].

**Figure 1:**
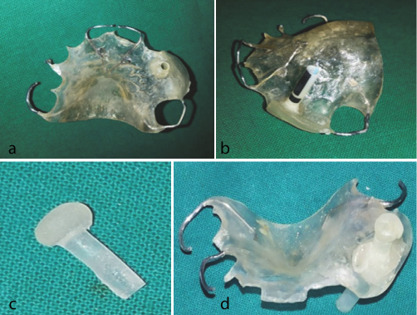
**a and b:** Modified surgical stent, **c:** Acrylic stopper, **d:** Modified surgical stent with acrylic stopper in situ

The purpose of this article is to report two cases of odontogenic cyst in the mixed dentition period, treated with a modified decompression technique (Figure 1a, b, c, d), which increased convenience and enhanced patient compliance to treatment.

## Case Presentation

### Case Report 1

**Figure 2:**
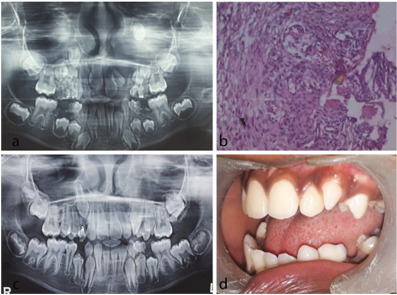
Case 1, **a:** The panoramic radiograph showing radiolucent lesion, **b:** Photomicrograph of radicular cyst. (400× magnification, H&E stain), **c:** 42 months postoperative panoramic radiograph, **d:** Postoperative intra oral photograph

A 7-year-old boy reported to the Department of Pediatric Dentistry with the primary complaint of pain and swelling in the upper left canine region over the last month. The patient experienced moderate pain during mastication lasting for 20-30 minutes after the onset. Upon clinical examination, diffuse extra-oral swelling was observed in the infraorbital region extending from the lower border of eyelid to the ala tragus line and from the corner of nose to the outer canthus level of the left eye. On palpation, the swelling was bony hard and tender. An intraoral examination revealed a cortical plate expansion from the maxillary left permanent incisor to primary first molar. The root piece of the deciduous left canine, grossly carious first and second left primary molars were noted. A panoramic radiograph revealed a well- defined periapical radiolucency measuring about 1.5cm×2cm around the periapical region of primary left canine with the successor tooth bud placed at a higher level than the contralateral permanent canine tooth bud (Figure 2a). Fine needle aspiration cytology of the cyst revealed a straw-colored fluid, which was abundant in polymorphonuclear neutrophils and red blood cells. Based on the clinical findings, radiographic investigation and fine needle aspiration cytology results, the diagnosis of radicular cyst was made. The decision was made to extract the left primary canine, and the first and second primary molars, followed by the curettage and decompression of the cyst. An extension through the extraction socket to the body of cyst was achieved with the help of a periosteal elevator. A soft tissue specimen measuring 1.5×0.5×0.25cm was removed during the curettage and the diagnosis of a radicular cyst was confirmed histopathologically (Figure 2b). Cystic lesion patency was maintained through the extraction socket with tightly packed Betadine soaked gauze. Patient was instructed to report every alternate day for change of dressing. During subsequent appointments, patient experienced severe pain and bleeding during fresh medicated gauze packing. This led to noncompliance from the child. Parents also showed reluctance for frequent visit to the hospital. Therefore, an alternative approach, using a modified surgical stent, was conceived in order to make the child more comfortable and reduce visit frequency. An alginate impression of the upper arch was made. A Hawley’s appliance was constructed. Approximately 4 mm hole was made through the acrylic plate over the extraction socket. A nasotracheal tube (3mm, Portex Tracheal Tube, Smiths Medical International Ltd., Hythe, Kent, UK) containing an embedded radio opaque marker within its walls, was passed through the plate and the extraction socket into the cystic lesion until resistance was felt. The tube was then attached to the Hawley’s appliance with cold cure acrylic (Figure 1a, b). A panoramic radiograph confirmed its position and extent in situ. Finally, an acrylic stopper was prepared to seal the tube during food consumption (Figure 1c, d). Patient’s parents were taught to irrigate the lesion regularly through the tube with copious amount of Betadine solution to reduce surgical site infection in accordance with a Cochrane systematic review [ 5 ]. Based on radiographic assessment, the length of the tube was frequently trimmed as the permanent canine erupted over a three and a half year period (Figure 2c, d). Overall, our modification allowed for the uninterrupted drainage of cystic fluid into the oral cavity, which led to the decompression of the cystic lesion. Patient had to report to hospital once or twice in 3 months for radiographic assessment and tube trimming. This was much lesser frequency compared to alternate day visit required for medicated gauze packing method. Once the permanent canine was closer to eruption, patient discontinued the wearing of the appliance. A total 42 months follow up was maintained until the permanent canine erupted into the oral cavity.

### Case Report 2

**Figure 3:**
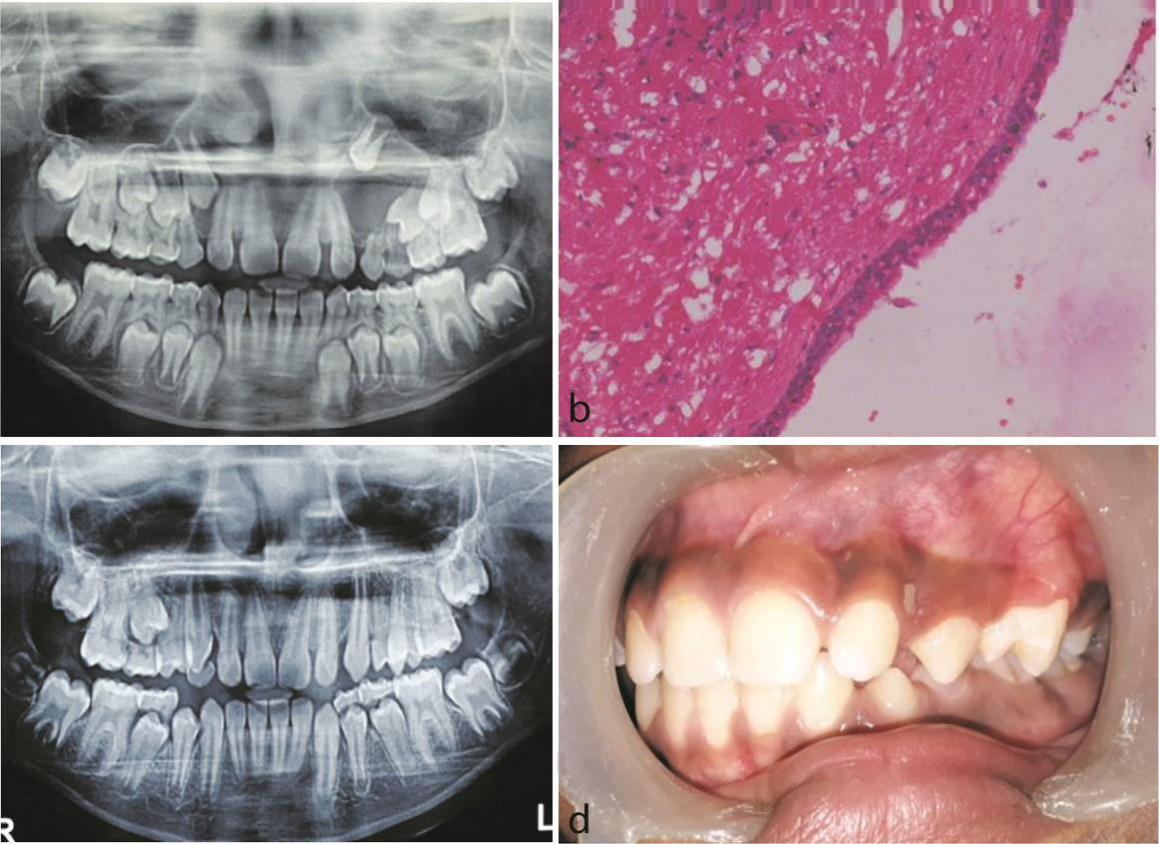
Case-2, **a:** The panoramic radiograph showing unilocular radiolucency, **b:** Photomicrograph of dentigerous cyst (400× magnification, H&E stain), **c:** 28 months postoperative panoramic radiograph, **d:** Postoperative intra oral photograph

An 11-year-old boy visited the Department of Pediatric Dentistry with primary chief complaint of asymptomatic swelling in the upper left canine region over a 3-monthperiod. Upon clinical examination, diffuse extra oral swelling was observed in the left infraorbital region extending from the lower border of eyelid to the ala tragus line and from the corner of the nose to the outer canthus level of the left eye. Upon palpation, the swelling was bony hard and an intraoral examination revealed a buccal and palatal cortical plate expansion from the maxillary left permanent lateral incisor to the left primary second molar. The teeth in this region did not exhibit any sign of dental injury. A panoramic radiograph revealed a well- defined unilocular radiolucency measuring 2cm×2cm, which extended from the lateral border of the left lateral incisor to the mesial border of the first left premolar tooth bud and surrounded the root of primary left maxillary canine. The succedaneous tooth germ of permanent canine was located higher compared to its counterpart on the right side (Figure 3a). Decompression was planned and the extraction of the maxillary left primary canine was carried out in order to gain access to the cystic lesion. The soft tissue scraped from the cystic lining was sent for histopathological investigation (Figure 3b). Based on the clinical, radiographic, and histological findings the lesion was diagnosed as dentigerous cyst. Consequently, modified surgical stent was considered after discussion with parents.

The decision of utilizing modified surgical stent was based on positive outcome of case study 1 as well as parental reluctance for regular visits to hospital. As described in case study 1, Hawley’s appliance was constructed from an alginate impression of the upper arch. A nasotracheal tube (size-3) was passed through the hole of the plate and the extraction socket into the cystic lesion until resistance was felt. The tube was then attached to the Hawley’s appliance with cold cure acrylic. The panoramic radiograph confirmed its position and extent in situ. An acrylic stopper was prepared to seal the tube during food consumption. Patient’s parents were taught to irrigate the lesion regularly through the tube with copious amount of Betadine solution. The length of the tube was frequently trimmed as the permanent canine erupted over two-and-a-half-year period. This uneventful eruption of the successor canine in its proper location again confirmed the usefulness of our appliance in management of odontogenic cysts in mixed dentition (Figure 3c, d). A follow up for28months was maintained.

## Discussion

The treatment choice in the management of odontogenic cysts in the mixed dentition period depends upon the size and location of the cyst, the bone integrity of the cystic wall and its proximity to vital structures. Masuda et al. [ 6 ] reported that in young children, enucleation should be avoided if the cyst is in close proximity to the developing tooth germ and other anatomical structures. Instead, marsupialization is recommended [ 6 ]. Many surgeons recommend enucleation following marsupialization in order to remove the pathological cystic lining and prevent the recurrence of the cyst [ 2 , 4 ]. However, extensive research has shown that marsupialization/ decompression alone is a sufficient method of treatment of large jaw cysts, especially in young children, because it facilitates good bone regeneration and normal eruption of permanent tooth [ 2 , 4 , 6 - 12 ]. In marsupialization technique, a part of the cystic lining is removed to create a surgical window to expose the cystic lesion to the oral cavity. This is followed by suturing the exposed cystic boundaries to the adjacent mucosa, whereas in the decompression technique, a drain is placed in the lesion, which maintains communication between oral cavity to cystic lesion. This decreases the intracystic pressure and results in bone formation. The main difference between these two techniques is the use of any device or drain instead of suturing of cystic lining to adjacent mucosa to prevent the closure of the mucosa. In both the techniques, a decrease of the intraluminal pressure/volume helps in resolving the cystic lesion [ 4 ].

In our cases, there was a close proximity of the tooth bud to cystic lining. Enucleation would have damaged the underlying tooth bud. Hence, we preferred to undertake decompression. The surgical procedure in our patients included the extraction of the involved deciduous tooth, the collection of incised pathological tissue for microscopic examination, and the establishment of an access point through the extracted socket into the body of cystic lesion. In both described cases, good bone healing with a normal eruption of the displaced permanent tooth was subsequently observed. The main purpose behind marsupialization /decompression is to make an opening or create an adequate window on the outer wall of cyst through which the cystic content is evacuated. This leads to the gradual ossification and size reduction of the cyst to an extent that the cystic lining becomes continuous with the oral epithelium [ 3 , 7 ]. This suggests that decompression within a bony cyst may dramatically promote the active growth potential of bone and the eruption of the tooth germ in the mixed dentition period. Various authors have reported successful results with different decompression technique. Oliveros-Lopez et al. [ 11 ] in their study effectively utilized decompression tube passing through surgically induced bony window over cystic wall. CarvanoandLuna [ 12 ] in their case series used a transalveolar silicone drain for decompression of dentigerous cyst in mixed dentition period. Nawaz et al. [ 13 ] reported the use of simple multipurpose acrylic splint in rehabilitation of radicular cyst in mixed dentition. Ziccardi et al. [ 14 ] in their case report used a simple fenestration technique for management of large dentigerous cyst.

However, the main drawback of decompression in children is patient compliance as it involves frequent clinical visits and prolonged treatment till the eruption of successor. Principally, the patency of the cystic lumen is maintained either by tightly packing sterilized gauze or by means of silicon drain or decompression tube, which requires frequent dressing changes and cystic cavity irrigation by clinician [ 3 , 11 - 12 ]. During a dressing change, a patient may experience extensive pain and fresh bleeding. Although our children reported moderate pain during change of gauge, it was sufficient enough to make them highly uncooperative in subsequent appointments. Our patient’s parents also showed reluctance for frequent visits. To overcome these shortcomings, we utilized a Hawley’s appliance to provide a stable template for the nasotracheal tube and to maintain continuous drainage from the cystic lesion into the oral cavity. The unique design of the appliance not only decompressed the cyst but also maintained the space for the eruption of successor tooth. The advantage of our design was its easy removal and insertion with minimal discomfort. Patients showed remarkable acceptance once they understood the path of insertion and removal of the appliance. They were able to maintain good oral and appliance hygiene. During food consumption, an acrylic stopper was inserted into to the oral end of the tube. This prevented the food, getting lodged into the tube. Our appliance reduced the number of visits and improved compliance and cooperation of our patients. Moreover, the easy fabrication of the appliance makes modified surgical stent in marsupialization an effective option. Both the patients gave a satisfactory feedback on the acceptability of the appliance in their feedback forms post treatment.

The authors certify that they have obtained all appropriate patient consent forms. In the form, the patients have given their consent for their images and other clinical information to be reported in the journal. The patients understand that their names and initials will not be published and due efforts will be made to conceal their identity, but anonymity cannot be guaranteed.

## Conclusion

Modified surgical stent is an effective and conservative approach of marsupialisation for large odontogenic cysts in mixed dentition. This appliance reduces the frequent clinical visits, maintains space for permanent dentition in children.

## Conflicts of Interest

 There are no conflicts of interest.
